# Covert Infection of Insects by Baculoviruses

**DOI:** 10.3389/fmicb.2017.01337

**Published:** 2017-07-17

**Authors:** Trevor Williams, Cristina Virto, Rosa Murillo, Primitivo Caballero

**Affiliations:** ^1^Instituto de Ecología AC Xalapa, Mexico; ^2^Bioinsecticidas Microbianos, Instituto de Agrobiotecnología, Consejo Superior de Investigaciones Científicas, Universidad Pública de Navarra Mutilva, Spain; ^3^Laboratorio de Entomología Agrícola y Patología de Insectos, Departamento de Producción Agraria, Universidad Pública de Navarra Pamplona, Spain

**Keywords:** mixed-mode transmission, Lepidoptera, latency, sublethal disease, persistent infection

## Abstract

Baculoviruses (*Baculoviridae*) are occluded DNA viruses that are lethal pathogens of the larval stages of some lepidopterans, mosquitoes, and sawflies (phytophagous Hymenoptera). These viruses have been developed as biological insecticides for control of insect pests and as expression vectors in biotechnological applications. Natural and laboratory populations frequently harbor covert infections by baculoviruses, often at a prevalence exceeding 50%. Covert infection can comprise either non-productive latency or sublethal infection involving low level production of virus progeny. Latency in cell culture systems involves the expression of a small subset of viral genes. In contrast, covert infection in lepidopterans is associated with differential infection of cell types, modulation of virus gene expression and avoidance of immune system clearance. The molecular basis for covert infection may reside in the regulation of host–virus interactions through the action of microRNAs (miRNA). Initial findings suggest that insect nudiviruses and vertebrate herpesviruses may provide useful analogous models for exploring the mechanisms of covert infection by baculoviruses. These pathogens adopt mixed-mode transmission strategies that depend on the relative fitness gains that accrue through vertical and horizontal transmission. This facilitates virus persistence when opportunities for horizontal transmission are limited and ensures virus dispersal in migratory host species. However, when host survival is threatened by environmental or physiological stressors, latent or persistent infections can be activated to produce lethal disease, followed by horizontal transmission. Covert infection has also been implicated in population level effects on host–pathogen dynamics due to the reduced reproductive capacity of infected females. We conclude that covert infections provide many opportunities to examine the complexity of insect–virus pathosystems at the organismal level and to explore the evolutionary and ecological relationships of these pathogens with major crop and forest pests.

## Introduction

Baculoviruses are large occluded dsDNA viruses that infect insects. The collapse of insect outbreaks in some species is associated with epizootics of baculovirus disease suggesting that these pathogens can regulate insect populations (Anderson and May, 1980; Cory and Myers, 2003; Elderd et al., 2013). In several cases these viruses have been developed for use as biological insecticides for the control of pests of forests and agricultural crops (Moscardi, 1999), or as expression vectors in biotechnological applications (Kost et al., 2005). In the present review we examine the evidence for covert infection of insects by baculoviruses, the similarities between baculoviruses and persistent virus infections in vertebrates, the relationship between virulence and transmission strategy, and the role of covert infection in the ecology of baculoviruses and their hosts.

**FIGURE 1:**
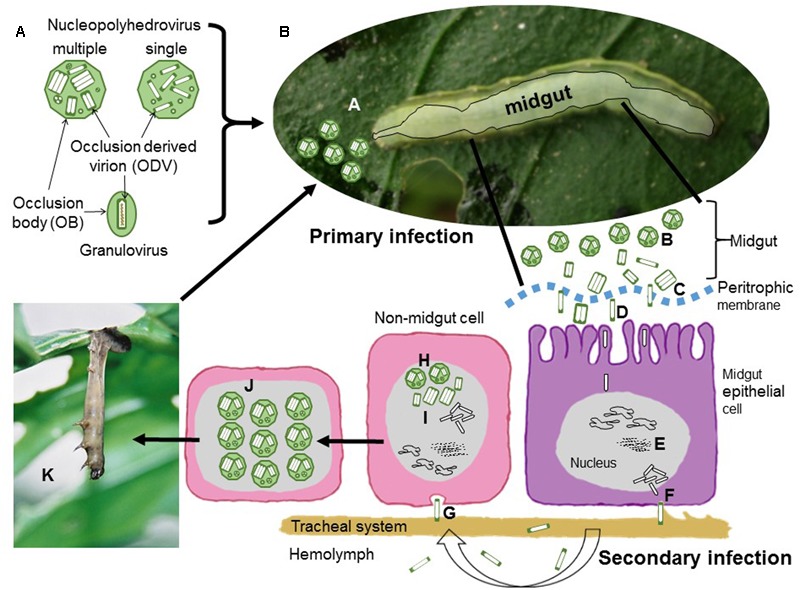
Schematic representation of baculovirus structure and infection cycle. **(A)** Nucleopolyhedrovirus occlusion bodies (OBs) are polyhedral proteinaceous bodies, mainly comprised of crystalline polyhedrin that surrounds occlusion derived virions (ODVs). The ODVs contain either a single nucleocapsid (single type) or between one and several nucleocapsids (multiple type) in each ODV. For granuloviruses the OB is granule-shaped and contains a single ODV with a single nucleocapsid surrounded by the crystalline protein granulin. In all cases each nucleocapsid contains a single viral genome. **(B)** Sequential steps of nucleopolyhedrovirus transmission and replication. During primary infection, (A) OBs are ingested during feeding on contaminated foliage. (B) OBs are solubilized in the insect midgut and release ODVs that pass through the peritrophic membrane (C) and fuse with the microvilli of midgut epithelial cells (D). Nucleocapsids travel to the nucleus where they release the viral genome to initiate replication. (E) Virus replication occurs in virogenic stroma. Progeny nucleocapsids assemble and bud through the basal membrane (F) during which they acquire an envelope containing GP64 or F fusion protein present in the virus-modified cell membrane. During the secondary phase of infection these budded virions (BVs) disperse in the hemolymph or along the cells of the insect traqueal system (traqueoblasts) to spread the infection to the cells of other tissues in the insect. (G) BVs enter cells by endocytosis and replicate in the nucleus. Newly assembled nucleocapsids (H) may bud out of the cell or may be enveloped to form ODVs that are occluded into OBs (I). At the end of the infectious cycle OBs accumulate in the nucleus (J). Upon death the larvae typically hang from the uppermost leaves of the host plant (K), the larval tegument ruptures and releases OBs that contaminate foliage for further cycles of horizontal transmission.

The current classification of the family *Baculoviridae* involves four genera: *Alphabaculovirus* comprising nucleopolyhedroviruses of Lepidoptera, *Betabaculovirus* comprising granuloviruses of Lepidoptera, *Gammabaculovirus* comprising nucleopolyhedroviruses of sawflies (Symphyta) and *Deltabaculovirus* comprising nucleopolyhedroviruses from mosquitoes (Diptera) (Herniou et al., 2012). The biology and ecology of these viruses is intimately related to their structure (**Figure 1A**). Rod-shaped nucleocapsids, each containing a single circular genome, are enveloped, singly or in groups, by a membrane to form virions that are occluded by a protein matrix, which forms the occlusion body (OB). The OB protects the virions, known as occlusion derived virions (ODVs), from environmental factors. These viruses can be readily distinguished into two groups: nucleopolyhedrovirus and granuloviruses that clearly differ in the structure of their OBs (Adams and Bonami, 1991). Nucleopolyhedroviruses have polyhedral OBs (0.5–10 μm), mainly comprising crystalline polyhedrin protein, which occlude large numbers of virions. In contrast, granuloviruses have smaller granule-like OBs (∼0.4 μm), that mainly comprise granulin protein. Each granulovirus OB contains a single virion.

In phytophagous Lepidoptera horizontal transmission occurs when OBs are consumed on contaminated foliage (**Figure 1B**). The OBs break down in the alkaline midgut, releasing ODVs that infect midgut cells. Following replication in midgut cells individual nucleocapsids bud out of the cell and these budded virions (BVs) disperse to infect other cells during the systemic phase of infection. Later, nucleocapsids are retained in the cell and become enveloped in ODVs and occluded to form OBs. Following death, large numbers of OBs are released from the insect cadaver for the following round of horizontal transmission. In sawflies, mosquitoes and some granuloviruses of Lepidoptera, infection is restricted to the larval midgut and the main mechanism for horizontal transmission involves fecal contamination of the local environment through disease associated diarrhea (Federici, 1997; Becnel, 2007; Arif et al., 2011).

### Replication Strategy

The baculovirus replication strategy involves a series of temporally coordinated events that begin when the infecting nucleocapsids release the viral genome into the nucleus (Rohrmann, 2013). During the first ∼6 h post-infection, the host RNA polymerase II transcribes immediate-early viral genes (*ie-0, ie-1, ie-2, pe38*) that are expressed in the absence of any other viral proteins and which encode transcription factors and delayed-early genes that promote genome replication and the expression of late genes and block apoptosis. The late genes (6–12 h post-infection) include a virally encoded DNA polymerase for genome replication, a virally encoded RNA polymerase, structural proteins and a range of late expression factors (*lefs*) involved in genome replication and transcription, as well as many genes with auxiliary functions. Viral DNA replication is concurrent with the expression of structural components necessary for the assembly of new nucleocapsids at 6–24 h post-infection and ODVs and OBs at 18–72 h post-infection. These temporal gene classes are mainly coordinated through DNA promoter elements (Rohrmann, 2013). Baculovirus infection induces a plethora of changes in host cell functions including cytoskeletal remodeling, cell cycle arrest, modulation of cellular stress responses and marked changes in cellular metabolism (see Monteiro et al., 2012 for a detailed review).

### Mixed-Mode Transmission

Baculoviruses adopt a mixed-mode transmission strategy involving both horizontal and vertical transmission that is common across a broad range of viruses, parasites, symbionts, and microbiota (Ebert, 2013). Horizontal transmission is usually risky if susceptible hosts are rare, while vertical transmission is safer, but is constrained by host survival and reproductive success. As a result, horizontal transmission is selectively advantageous at high host densities, whereas vertical transmission is favored in low density host populations. In the case of baculoviruses, these transmission strategies generally exclude one another because the production of massive numbers of OBs for horizontal transmission results in host death prior to the adult stage. Under such a trade-off, experimental studies on viruses of bacteria and plants indicate that selection for fitness gains accrued through one route reduce the importance of the alternative route (Turner et al., 1998; Stewart et al., 2005).

Through the examples provided in the following sections it will become evident that mixed-mode transmission involving long-lived viral OBs in the environment and vertical transmission from infected adult insects to their offspring has key implications for the persistence, spatial dispersal and genetic diversity of insect baculoviruses (Cory, 2015). Most of these examples come from alphabaculoviruses and betabaculoviruses of phytophagous insects, as the processes of transmission and replication are poorly understood in gammabaculoviruses and deltabaculoviruses of sawflies and mosquitoes, respectively.

## Covert Infection

One aspect of the disease dynamics of baculoviruses which is increasingly attracting attention, is the maintenance of the virus in the host population when opportunities for horizontal transmission are limited. As vertical transmission requires host reproduction, it is clear that this route is only possible for viruses that can adopt a low virulence host utilization strategy. In evolutionary terms virulence is a measure of the harm suffered by an infected host, often reflected in decreased survival or reproduction (this should not be confused with the definition used by invertebrate pathologists who use virulence to describe the disease-producing power of the pathogen; Shapiro-Ilan et al., 2005). As host resources are used by viruses for their replication, the production of progeny viruses has to be balanced against the predicted likelihood of horizontal or vertical transmission.

**FIGURE 2:**
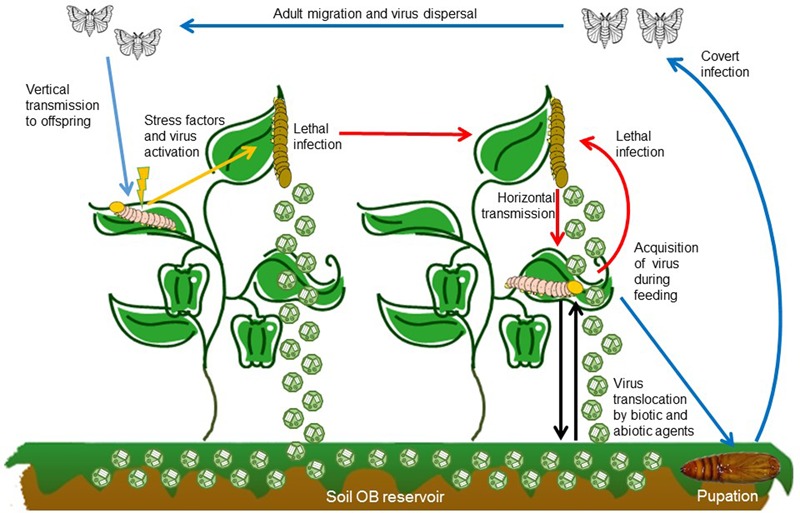
Baculovirus transmission routes, mode of infection and dispersal pathways in the environment. After larvae ingest OBs while feeding on contaminated foliage a portion of the infected individuals develop lethal disease and release OBs onto the host plant where they can be transmitted to a susceptible host (red arrow). OBs on foliage are also washed by rainfall into the soil, from which they can be transported back to plants by biotic and abiotic factors (black arrows). Alternatively, insects that consume OBs but survive may continue to develop, pupate and emerge as covertly infected adults (blue arrows). These adults can disperse before laying eggs and passing the infection to their offspring. Vertical transmission can be sustain over several generations until some elicitor or stress factor triggers (orange arrow) the covert infection into lethal disease which returns to the horizontal transmission cycle (red arrows).

For baculoviruses, low virulence is associated with covert infection. Insect larvae that ingest OBs but do not die can continue their development and emerge as covertly infected adults (**Figure 2**). Covert infections (also known as inapparent, sublethal, silent or occult infections) are characterized by the absence of visible signs of disease. This type of infection has clear parallels with infections involving low level replication by viruses such as polyomaviruses in vertebrates or non-productive latent infections by herpesviruses, retroviruses, and bacteriophages.

In line with virulence theory, vertically transmitted genotypic variants of SeMNPV from field-collected adults of *Spodoptera exigua* were significantly less pathogenic and less virulent (slower killing) than horizontally transmitted variants isolated from the soil (Cabodevilla et al., 2011a). Genome sequence analysis allowed the identification of genes that differed between vertically and horizontally transmitted genotypes (Thézé et al., 2014). Of these, four genes (*se4* [*hoar*], *se5, se76* [*cg30*], and *se129* [*p26*]) were shown to reduce OB pathogenicity using a bacmid-based system to produce deletion mutants, whereas speed of kill was unaffected except in the case of *se5* of unknown function, which was significantly extended in the deletion mutant (Serrano et al., 2015).

At present there is no evidence that covertly infected insects release OBs that could be transmitted horizontally. However, the baculovirus remains fully competent within the host and, at a certain moment, can be triggered to produce overt, lethal disease (Burden et al., 2006).

When the survival of the host appears to be threatened, opportunities for transmission sway in favor of horizontal rather than vertical routes necesitating a marked increase in the production of ODVs and OBs and a corresponding increase in virulence. The covert infection itself may be productive or non-productive depending on whether the virus adopts a persistent or latent mode of infection.

### Persistent vs. Latent Infections

There are two types of covert infection that differ in their replication strategy and pathology. Persistent infections involve low levels of virus replication – a range of viral genes are expressed and virions can be produced. Pathological effects may also be observed that can be costly to host fitness. We will describe these effects as sublethal disease. Latent infection, in contrast, does not involve the production of virions and a limited number of virus genes are expressed to maintain latency (Chao et al., 1998; Kane and Golovkina, 2010).

Analysis of the studies performed over the past 25 years since the widespread adoption of molecular tools (Supplementary Table 1) reveals a number of trends.

(i) Covert infections have been detected in both laboratory colonies and natural populations of insects infected by viruses from all four genera in the *Baculoviridae*, but have been studied most frequently in nucleopolyhedroviruses of Lepidoptera (genus *Alphabaculovirus*).
(ii) PCR-based techniques and more recently qPCR, have been employed to detect covert infections and estimate their prevalence. PCR-based detection has targeted genes that are required for genome replication (*dpol, lef-8*), or the assembly of structures such as viral capsids (*vp39, vp80*) or OBs (*polh, gran*). Detection of these transcripts would be consistent with low-levels of virion production in persistently infected insects although this has not been demonstrated explicitly.
(iii) Of the 36 studies listed in Supplementary Table 1, 10 were focused on determining the presence or absence of covert infection, whereas the remaining studies attempted to estimate the prevalence of infection – and over half of these studies indicated that covert infection could be present in 50% or more of the individuals in an insect population.

The sensitivity of the techniques used for detection of covert infection has generally increased, although it is difficult to draw firm conclusions due to the diversity of samples analyzed and variations in methodological procedures. The detection limits have fallen from approximately 0.1–10 pg genomic DNA, representing 100s or 1000s of genomes (Burden et al., 2002; Vilaplana et al., 2010; Kemp et al., 2011), to approximately 5–7 genomes per reaction using qPCR techniques (Virto et al., 2013; Graham et al., 2015), or ∼35 genomes using RT-PCR (Cabodevilla et al., 2011a).

Clearly, the results of PCR-amplification of viral DNA will depend on the ongoing genome replication activity within each cell, while the results of RT-PCR reflect the transcriptional activity of the selected target gene. The selection of a structural protein gene or a gene involved in genome replication or transcription can consequently generate quite different estimates of the prevalence of covert infection, and also provides information on the replication status of the infection (Martínez et al., 2005).

Unlike persistent infections, the evidence for latent infection in insects is very limited. As new generation sequencing and transcriptomic techniques begin to be applied to the study of insect viruses, evidence for the production of latency-associated transcripts (LATs) and virus integration into the host genome is appearing from cell culture systems that are analogous to the mechanisms employed by herpesviruses and other viruses with persistent infection strategies (discussed in the following sections).

## Vertical Transmission

The passage of baculovirus to a subsequent generation comprises both the transovarial and transovum pathways. Transovarial transmission involves the process of virus passing from the maternal parent to progeny embryos within the eggs, whereas the transovum route involves contamination of the exterior egg surface with viral particles that infect neonate larvae as they ingest the chorion during hatching (eclosion) (Kukan, 1999; Cory and Myers, 2003). In Lepidoptera the usual procedure to distinguish between these transmission pathways consists of surface decontamination by virucidal formalin or hypochlorite treatment. External disinfection of eggs has been observed to reduce the prevalence of disease by at least 10-fold in many insect–virus pathosystems (Kukan, 1999), and is regularly used during insect rearing to reduce the risk of outbreaks of baculovirus disease in the colony.

In systems involving the nucleopolyhedroviruses of the silkworm (*Bombyx mori*), gypsy moth (*Lymantria dispar*), the beet armyworm (*S. exigua*) and the African armyworm (*Spodoptera exempta*), surface decontamination had little effect on the prevalence of spontaneous virus disease or covert infection in the offspring, indicating transovarial transmission in these species (Myers et al., 2000; Khurad et al., 2004; Vilaplana et al., 2008; Virto et al., 2017b).

Males have also been implicated in the vertical transmission of baculoviruses. The presence of covert infection in the offspring of matings between uninfected females and infected males, and vice versa, indicated that both sexes were involved in vertical transmission (Burden et al., 2002; Cabodevilla et al., 2011b; Virto et al., 2013). Maternal transmission of SeMNPV was approximately twice as efficient as the paternal route, not only because females transmitted infections to a higher proportion of their offspring, but also because viral loads were higher in the offspring of infected mothers compared to those of infected fathers (Virto et al., 2013).

In line with these findings, viral transcripts have been detected in the testis and ovaries of sublethally infected *Plodia interpunctella* adults (Burden et al., 2002), and OBs have been observed directly in the reproductive organs of both sexes in *B. mori* (Khurad et al., 2004).

Nucleopolyhedroviruses of mosquitoes and sawflies appear to be capable of indirect vertical transmission by contaminating the offspring’s environment with OBs from the maternal gut. In the European pine sawfly, *Neodiprion sertifer*, the meconium voided by the female following adult emergence from the puparium contained viable OBs and was responsible for initiating infections in offspring (Olofsson, 1989). Likewise, a low level of vertical transmission (∼5%) was detected in *Culex nigripalpus* egg rafts that appear to have been contaminated with OBs released from adult female meconium or possibly adult feces (Becnel et al., 2003).

Vertical transmission also has implications for viral genetic diversity. Genetic variation in baculovirus populations favors horizontal transmission due to heterogeneity in host susceptibility to infection (Hudson et al., 2016) and interactions among virus genotypes that increase OB infectivity and OB production in infected insects (Clavijo et al., 2009; Barrera et al., 2013; Bernal et al., 2013). Indeed, alphabaculovirus ODVs and OBs are genotypically diverse and form collective infectious units that ensure horizontal transmission of multiple viral genotypes (Sanjuán, 2017). However, vertical transmission likely represents a genetic bottleneck for baculoviruses. For example, reduced diversity was observed in a laboratory study on *Spodoptera frugiperda* in which a vertically transmitted isolate differed in restriction endonuclease profile characteristics compared to the genotypically diverse wild-type isolate used to inoculate the parental generation (Fuxa et al., 1994). Insects infected with the vertically transmitted isolate experienced an elevated prevalence of virus-induced mortality during larval and pupal development (Fuxa and Richter, 1991). OBs were also observed in the cells of these insects but many of the OBs did not contain ODVs (Fuxa et al., 1992). From the host’s perspective, limiting variation in a vertically transmitted virus means that host offspring are less likely to have to face issues of evolving virulence among viral genotypes, as genotypic diversity favors increased virulence within infected hosts (Frank, 1996b). Moreover, uniparental vertical transmission further restricts viral diversity in the offspring as it prevents mixing of viruses in the maternal and paternal lines of descent (Frank, 1996a). These concepts are ripe for testing in baculovirus pathosystems.

## Maintenance of Covert Infections

Insects have developed a diversity mechanisms to protect themselves from, or minimize the impact of viruses, involving both the acquired and innate immune response (Sparks et al., 2008). Induced host defense mechanisms include the release of antimicrobial peptides (Cheng et al., 2006), phagocytosis (Narayan, 2004), cell apoptosis (Clem, 2015), and sloughing of infected midgut cells (Washburn et al., 2003; Haas-Stapleton et al., 2005). Studies on host immune response to viruses often use hemocyte counts and phenyloxidase activity in the hemolymph as indicators of immune function (Klemola et al., 2007; Shikano et al., 2010; Silva et al., 2013). In some cases not all aspects of the immune functions respond equally to immune stimuli, so that trade-offs may be observed between different aspects of immune function depending on the nature of each potential threat (Shlichta and Smilanich, 2012; Wilson and Cotter, 2013).

Viral strategies for maintaining covert infections include the selection of specific cell types for the maintenance of viral genomes, the modulation of viral gene expression, and the avoidance of clearance by the host immune system (Kane and Golovkina, 2010). The selection of cell types and tissue tropism of baculoviruses in covertly infected insects remains unclear. PCR-based analyses of larvae almost invariably involve extraction of nucleic acids from the entire body, whereas studies on adults have focused on the contents of the abdomen (Hughes et al., 1997; Virto et al., 2014), or gonadal tissue (Burden et al., 2002). In a detailed study Graham et al. (2015) determined that the highest titers of SpexNPV in *S. exempta* adults were present in the wings, head and legs, while low titers were found in the thorax and abdomen. The abundance of virus genomes in the extremities of *S. exempta* adults may reflect the immunoprivileged nature of these structures in moths, with both: (i) low circulation of hemocytes and hemolymph-associated antimicrobial factors, and/or (ii) the presence of neurons and ganglia that are metabolically less active and less prone to hemocyte infiltration than most tissues present in the thorax or abdomen (Graham et al., 2015). This is analogous to the selection of sensory neurons for the indefinite persistence of herpes simplex virus (HSV), or renal epithelial cells persistently infected by polyomaviruses such as SV40, JC or BK viruses (Kane and Golovkina, 2010).

Another key aspect of the maintenance of covert infection is the role of the viral apoptosis suppressor proteins, such as P35, IAPs 1–5, P49 and Apsup, which block cellular apoptosis or global protein synthesis shutdown following infection (Ikeda et al., 2013). Expression of viral early genes triggers apoptosis but P35 synthesized during the early and late phases of infection localizes to the cytoplasm and inhibits effector caspases by direct and irreversible binding. The other anti-apoptotic proteins have different modes of action (Ikeda et al., 2013).

Cell culture studies indicated that antiapoptosis genes can modulate covert infection and the activation of such infections to a lytic (lethal) state. For example, the inhibitor of apoptosis gene (*iap*) from a granulovirus was shown to be able to substitute the function of the LAT in a HSV model (Jin et al., 2005). In contrast, the deletion or modification of the *p35* baculoviral suppressor of apoptosis in AcMNPV resulted in a sublethal infection of *S. frugiperda* (Sf9, Sf21) cells that released low levels of virus progeny into the culture medium (Lee et al., 1998). When the *p35* gene was transfected under the control of an immediate-early promoter virus titers increased to near wild-type values. Persistently infected cells were resistant to superinfection by the wild-type virus but transfection of *p35* stimulated virus activation and prolific production of virus progeny.

Recently, a functional serpin (serine protease inhibitor) was found to be encoded by a nucleopolyhedrovirus that infects a saturnid moth, *Hemileuca* sp. (Rohrmann et al., 2013). The Hesp018 protein was shown to inhibit prophenoloxidase activation in hemolymph, suggesting that it may contribute to supressing the host humoral immune response. Expression of the serpin in a recombinant nucleopolyhedrovirus also resulted in accelerated production of BVs in cell culture and a fourfold reduction in the quantity of OBs required for lethal infection of *Trichoplusia ni* larvae, indicating a role in modulating virulence (Ardisson-Araujo et al., 2015).

### Latent Infection

In latent infection, two main scenarios have been envisaged regarding the persistence of viral genomes within host cells: (i) The integration of the viral DNA into the genome of the host cell as a provirus structure, as occurs in bacteriophages and retroviruses, such as HIV (Lassen et al., 2004). Alternatively, partial integration of the viral genome into host chromosomes may occur, the best understood models for which involve human oncogenic viruses (Farazi and DePinho, 2006; Tashiro and Brenner, 2017). (ii) Viral genomes may be maintained as independent episomes associated with nuclear histones, as occurs in herpesviruses (Roizman and Whitley, 2013). Some herpesviruses may even persist simultaneously in both partially integrated and episomal forms (Morissette and Flamand, 2010).

Studies in insect cell culture systems have provided evidence of a latent infection and partial integration by baculoviruses involving between 13 and 20 virus genome copies in each infected cell (Hughes et al., 1994; Weng et al., 2009). In a study on SeMNPV, fragments of the viral genome were detected in *S. exigua* (Se301) cells following repeated passage of the virus. Latently infected cells did not produce virions although transcripts from the polyhedrin (*polh*) gene were detected. The infected cells were somewhat resistant to superinfection by SeMNPV, but their susceptibility to a heterologous virus (AcMNPV) appeared unaffected (Weng et al., 2009).

Deep sequencing of latently infected Se301 cells identified a set of 10 SeMNPV transcripts that were expressed during latency (Fang et al., 2016). An additonal six transcripts were detected by rapid amplification of cDNA ends (RACE). Importantly, these transcripts were present as chimeric fusion transcripts in combination with cellular genes, suggesting that a selection of viral genes had become integrated into the host genome. Of the total of 16 viral genes expressed in latency, three were late expression factors (*lef-2, lef-3, lef-6*), one was an apoptosis suppressor (*iap-2*), and the remainder had other functions including envelope fusion protein (F protein), ribonucleotide reductase small subunit, a DNA-binding protein (Ac25), a polyhedron envelope associated protein (*se11*), and a zinc finger protein suggested to be a transcriptional regulator or scaffold proten for ODV and budded virus assembly (*me53*), whereas the others were genes of unknown function (*se5, se44, ac18, ac19, ac34, ac68, ac69*), of which two (*se5, se44*) were unique to SeMNPV (Fang et al., 2016). These 16 transcripts were clustered in four equidistant groups on the SeMNPV genome, the functional importance of which is unclear.

### The Role of RNA Interference and Apoptosis in Covert Infection

The molecular basis for covert infection may quite likely reside in the regulation of host–virus interactions through the action of microRNAs (miRNA). These are small, non-coding, hairpin RNAs of ∼22 nucleotides produced by both hosts and viruses to regulate transcription and translation (Asgari, 2015). Following virus infection in insects the host antiviral responses are activated, including the production of small interfering RNAs (siRNAs) that target viral genes, viral transcripts or replication intermediates (Bronkhorst and van Rij, 2014), and numerous cellular miRNAs with diverse targets (Mehrabadi et al., 2013; Wu et al., 2016). For example, inhibition of the host miRNA known as *bantam* in AcMNPV-infected lepidopteran cells increased the expression of viral genes *lef-8, gp41* and *p10* and increased viral DNA replication, although final virus yields were not affected (Shi et al., 2016). To combat this type of response, viruses may produce their own miRNAs to target host and viral genes, or attempt to usurp host responses to their own advantage, so as to facilitate replication (Hussain and Asgari, 2014). Most of these studies have been performed using insect cell culture systems, although similar processes are believed to occur in whole insects.

For baculoviruses, miRNAs autoregulate replication and likely provide an extended temporal window for replication in order to maximize virion production without compromising host survival until shortly prior to death. For example, a miRNA (BmNPV-miR-3) regulates the P6.9 DNA binding protein gene and a number of other late virus genes, and allows the virus to escape the early innate immune response in silkworm larvae (Singh et al., 2014). In AcMNPV, a viral miRNA (AcMNPV-miR-1) suppressed the ODV-E25 (*ac94*) gene and facilitated the switch from budded virus to ODV production (Zhu et al., 2013, 2016). There are also examples of baculovirus miRNAs that target host genes. BmNPV-miR-1 targets the host *Ran* gene that encodes the Ran cofactor required for export of miRNA precursors from the nucleus to the cytoplasm by the Exportin-5 protein. This results in a total cellular reduction in host miRNAs which presumably reduces host interference, although it is not clear how viral miRNAs circumvent this process (Singh et al., 2012).

Host humoral responses may also be modulated by viral miRNAs. For example, miRNAs identified in the fatbody and midgut of nucleopolyhedrovirus (BmNPV) infected silkworms were predicted to target viral and host genes, four of which were related to insect immune function (Singh et al., 2010). The production of miRNAs capable of interfering with prophenyloxidase, hemolin (which modulates hemocyte aggregation and phagocytosis) and serine proteases (required for the activation of prophenyloxidase), points to intriguing potential mechanisms for avoiding clearance by the host immune system during latent or persistent infections.

The only known of example of miRNA involvement in latency in insect viruses comes from a study on a nudivirus (HzNV-1) in lepidopteran cells. This virus can establish latent infection in cells through expression of the persistent-associated gene 1, *pag1*. During latency *pag1* is the only viral transcript detected (Chao et al., 1998). Another viral gene, *hhi1* is capable of activating latent infections into productive and lethal infections, which normally occur in just a small fraction (<0.2%) of cells (Lin et al., 1999; Wu et al., 2010). For this, the *pag1* transcript was shown to be processed into two distinct miRNAs that targeted and degraded the *hhi1* transcript and were capable of inducing latency in HzNV-1 infected cells (Wu et al., 2011).

The production of the *pag1* transcript in HvNV-1 may be analogous to the mechanism that maintains latency in HSV, in which a LAT is continuously expressed whereas transcription of lytic genes is suppressed through their association with heterochromatin (Knipe and Cliffe, 2008). The LAT is a miRNA precursor that is processed into six miRNAs that inhibit cellular apoptosis and block the expression of early viral genes (Umbach et al., 2008; Shen et al., 2009).

Intriguingly, *p35* also functions as a suppressor of host RNA interference (RNAi) independently of its anti-apoptotic role (Mehrabadi et al., 2015). The mechanism of action of *p35* remains uncertain but appears to act downstream of Dicer-mediated cleavage of dsRNA, possibly by sequestering host siRNAs, or interfering with the function of Argonaute (Ago-2) protein or the assembly of the RNA-induced silencing complex (RISC) (Mehrabadi et al., 2015). Given the abundance and diversity of miRNAs produced by baculoviruses, numbering up to 48 in the case of *Spodoptera litura* nucleopolyhedrovirus (SpltNPV) (Kharbanda et al., 2015), it seems likely that viral miRNAs will soon be implicated in many aspects of the host–virus relationship, including different facets of covert infection.

## Factors that Favor Virus Activation

Covert infections were first proposed to explain the spontaneous outbreaks of baculovirus disease that occurred in apparently healthy insects (**Figure 2**). Early studies concluded that physiological stress was a major contributor to virus activation (Podgawaite and Mazzone, 1986). Specifically, overcrowded rearing conditions (Fuxa et al., 1999; Opoku-Debrah et al., 2013), marked changes in temperature or relative humidity (Fuxa et al., 1999; Kouassi et al., 2009), the ingestion of mildly toxic chemical compounds (Ilyinykh et al., 2004; Virto et al., 2017a), parasitism (Stoltz and Makkay, 2003), or changes in nutrient availability (David and Gardiner, 1965, 1966), have all been reported as potential activators of overt disease, although insect responses are often unpredictable. One report on granulovirus infection in *Pieris rapae* indicated that dehydration-induced activation of lethal disease could be atenuated to a non-lethal covert infection by switching larvae from a dessicated diet to a diet with a high water content (Biever and Wilkinson, 1978). This observation has yet to be verified in other insect baculovirus systems.

Stressors such as high temperatures and ultraviolet light are well-recognized to initiate genome derepression and expression of the lytic pathway in human HSV infections of sensory neurones (Roizman and Whitley, 2013), whereas DNA damage, hypoxia and exposure to cytotoxic tumor necrosis factors induce activation of the Z and R promotors that initiate the lytic cycle in Epstein-Barr virus-infected B memory cells in humans (Kenney and Mertz, 2014). These processes clearly reflect virus responses to threats of reduced survival in the infected host and seem to be common among pathogens with mixed-mode transmission. The ability to respond to changes in expected host survival and anticipated reproduction, and adopt the transmission pathway that will maximize the fitness derived from each infected host, provides a unique evolutionary advantage to the virus. As such, phenotypic plasticity in transmission strategy means that viruses with mixed-mode transmission can persist under a wider range of ecological conditions and at a higher prevalence than viruses that adopt strict single-mode transmission. That said, the complexity of epidemiological processes and the biological constraints acting on each host–virus pathosystem often hinder our ability to make clear predictions on transmission mode, even in simple systems (Ebert, 2013).

Perhaps the most consistent elicitor of virus reactivation is a challenge by a second pathogen (superinfection), often involving inoculation with heterologous viruses that have been isolated from insect species other than the host species under study (Longworth and Cunningham, 1968; Jurkovicova, 1979; Hughes et al., 1993; Fuxa et al., 2002; Cooper et al., 2003; Kouassi et al., 2009).

As infection by a second pathogen represents a major threat to host survival, the activation of overt baculovirus disease reflects a switch to a horizontal transmission strategy, as competition between viruses favors increased virulence (Frank, 1996b). Some support for this in baculoviruses comes from the observation that covertly infected larvae of *S. exigua* were 2–3-fold more susceptible to lethal superinfection as healthy conspecifics, when challenged with the same nucleopolyhedrovirus (SeMNPV) (Cabodevilla et al., 2011a,b). In contrast, simultaneous infection by SeMNPV and non-lethal iflaviruses (small RNA virus) tended to increase OB pathogenicity and vertical transmission in *S. exigua* (Carballo et al., 2017), possibly because the iflaviruses could improve their transmission by contaminating OBs produced in insects infected by both types of virus (Jakubowska et al., 2016). Despite the abundant circumstantial evidence for the activation of baculovirus covert infections, the molecular mechanisms underlying this process at the organismal level remain unclear.

## Sublethal Effects

Insects that do not die following the ingestion of viral OBs experience a number of adverse effects on aspects of their fitness (Rothman and Myers, 1996). These debilitating effects have their origins in three possible explanations: (i) a direct result of the pathological effects of the virus in the host, (ii) a result of the energetic costs incurred from mounting an immune response to suppress pathogen replication and, (iii) the result of host traits that are corrected with disease resistant phenotypes of individuals, i.e., those that are more likely to survive following ingestion of OBs (Myers and Kuken, 1995; Rothman and Myers, 1996; Bouwer et al., 2009). It is difficult to quantify the contribution of each of these effects to the overall fitness deficit observed in the survivors of a virus challenge, and in many cases sublethal effects may involve combinations of pathology, immune costs and resistant phenotype effects.

The role of host phenotype has begun to be examined by rearing siblings from egg masses or using insects such as tent caterpillars which live in sibling groups (families) that can be compared for within-group and between-group susceptibility to lethal infection and sublethal effects (Páez et al., 2015; Hudson et al., 2016; Myers and Cory, 2016). Indeed, susceptibility to nucleopolyhedrovirus varies across families so that the risk of mortality depends on virus isolate and family interactions in gypsy moth (*L. dispar*) larvae (Hudson et al., 2016).

Host phenotypes are very evident in some species of Lepidoptera. For example, in certain species rearing at high density induces phase polyphenism, a change from a pale to a dark melanic form (Goulson and Cory, 1995; Wilson and Cotter, 2009; Silva et al., 2013). This phenomenon is closely associated with increased investment in immune function and reduced susceptibility to pathogens, known as density dependent prophylaxis (Wilson and Cotter, 2009). This system could prove useful in examining the role of host phenotype on the relationship between covert infection and sublethal disease.

In general, the magnitude of sublethal effects observed in the survivors of a virus challenge rarely depends on the dose of OBs consumed. Sublethal effects also tend to be more pronounced in insects that are challenged in the later instars rather than early instars (Rothman and Myers, 1996; Myers et al., 2000). The survivors of a virus challenge often have extended development times in the larval and pupal stages (Goulson and Cory, 1995; Milks et al., 1998; Cabodevilla et al., 2011b), reduced pupal weight (a predictor of adult body weight) (Murray et al., 1991; Myers et al., 2000; Duan and Otvos, 2001; Matthews et al., 2002; Monobrullah and Shankar, 2008), and reduced female fecundity and egg fertility (Sait et al., 1994, 1998; Milks et al., 1998; Cabodevilla et al., 2011b). The preoviposition period was also significantly longer in virus-challenged survivors of *S. exigua* (Cabodevilla et al., 2011b), *S. exempta* (Vilaplana et al., 2008), and *Pieris brassicae* (Sood et al., 2010), so that female moths may be able to travel longer distances than healthy conspecifics before starting to lay eggs, resulting in increased dispersal of covertly infected progeny. Alterations in sex ratio in adult survivors of virus treatments in favor of females have been reported for *S. exigua* (Cabodevilla et al., 2011b), *Spodoptera littoralis* (Vargas-Osuna and Santiago-Alvarez, 1988; Scheepens and Wysoki, 1989), *Mamestra brassicae* (Goulson and Cory, 1995), and *Mythimna separata* (Patil et al., 1989), which is relevant if females are mainly responsible for host-mediated virus dispersal. Finally, an elevated metabolic rate of covertly infected *Helicoverpa armigera* larvae was taken as evidence of the metabolic costs of mounting an effective immune response (Bouwer et al., 2009). However, the causes underlying reduced fitness in the survivors of a virus challenge many be composite and difficult to dissect.

## Ecological Implications of Covert Infections

Covert infection in combination with mixed-mode transmission provides a mechanism by which the pathogen can survive when opportunities for horizontal transmission are scant, such as during periods of low host population densities, during diapause or non-overlapping generations. However, when host densities exceed a threshold that allows sustained horizontal transmission, the virus has the opportunity to reactive and produce patent disease that kills the host and releases progeny OBs for horizontal transmission (Cooper et al., 2003). This can trigger epizootics of disease in high density lepidopteran populations in field crops and forest habitats that rapidly reduce the host population to below the threshold density (Myers and Cory, 2016). Covert infection in highly mobile or migratory species also provides a mechanism for virus dispersal over large distances (Hostetter and Bell, 1985; Burden et al., 2003; Vilaplana et al., 2010) (**Figure 2**).

Transgenerational host–pathogen interactions can also convey benefits to the host as a defense against further infections (Jones et al., 2011). For example, the vertically transmitted bacterial symbiont, *Wolbachia*, provided protection against infection by a small RNA virus (*Dicistroviridae*) (Hedges et al., 2008). Similarly, infection of *Helicoverpa armigera* by a densovirus (*Parvoviridae*) appears to protect against a second infection by an alphabaculovirus or the bacterial pathogen *Bacillus thuringiensis* (Xu et al., 2014). Conversely, *S. exempta* larvae infected by *Wolbachia* were markedly more susceptible to SpexNPV than *Wolbachia*-free larvae (Graham et al., 2012).

At the population level, reduced fecundity of covertly infected insects was identified as the most likely cause for delayed recovery in populations of the Western tent caterpillar, *Malacosoma californicum pluviale*, that crashed following epizootics of alphabaculovirus disease (Cory and Myers, 2009). Unfortunately testing this hypothesis using molecular methods was problematic due the paucity of insects present in low density populations (Myers and Cory, 2016).

Population models make several clear predictions regarding the ecological impact of covert infections. In age structured insect populations covert infections were predicted to affect the periodicity and amplitude of population cycles (Bonsall et al., 2005). In a subsequent model, a low prevalence of covert infection was favored if opportunities for transmission varied, for example seasonally. In contrast, a high prevalence of covert infection was predicted under three specific scenarios: (i) when covert infection was due to host immune suppression, (ii) when covert infection directly improved the probability of transmission through patent lethal disease, and (iii) when covert infection protected against lethal infection by other pathogens (Sorrell et al., 2009). These models have provided clear predictions that can be tested empirically in laboratory and field populations.

It is worth noting that covert infections by baculoviruses have rarely been quantified because of a lack of interest in potential pathogens in insects that appear to be healthy (Okamura, 2016). Moreover, viral infections are rarely searched for in uncommon insects or non-pest species, so that covert infections in populations of uncommon hosts are likely to be overlooked unless systematic surveys are performed (Roy et al., 2009). Support for this idea comes from a study on a nucleopolyhedrovirus of the winter moth *Operophtera brumata*, in which two sympatric but less common moths were found to harbor covert infections and were likely to act as reservoir species for the winter moth virus (Graham, 2005). In general, the role of alternative host species in the persistence of baculoviruses has not been the subject of systematic investigation and this is an area that is overdue for study.

Finally, from a pest control perspective, sublethal effects in insect survivors after field application may be desirable and benefit pest control in subsequent generations as covertly infected insects might be more susceptible to a second virus application, so that effective pest control could be achieved with lower rates of OBs applications (Virto et al., 2017b).

## Future Perspectives

Baculoviruses share a number of common threads with viruses that cause persistent infections in vertebrates, namely selection of specific cell types for genome maintenance, modulation of viral gene expression, viral subversion of apoptotic pathways and avoidance of clearance by the host immune system (Kane and Golovkina, 2010). As next generation sequencing techniques become widely adopted we expect covert infections by baculoviruses to become more apparent and likely more diverse (Liu et al., 2011, 2015). Indeed, genetic diversity in covert infections is far from clear although preliminary evidence indicates that certain genotypes may be better adapted to non-virulent infection strategies (Cabodevilla et al., 2011a). From a mechanistic standpoint, the role of miRNAs in the persistence of covert infections is likely to become increasingly apparent over the coming decade and may even provide a means by which immune function costs and the pathological effects of sublethal disease can be dissected at both the individual and population levels. The study of baculovirus covert infections in insects provides a wealth of opportunities to understand the complexity of insect–virus pathosystems at the organismal level, test current aspects of evolutionary biology, such as virulence theory, and determine the ecological significance of these pathogens on major crop and forest pests.

## Author Contributions

CV, RM, TW, and PC surveyed and discussed the previous research, TW wrote the paper with support from CV, RM and PC, CV, RM and TW prepared the figures, PC and TW obtained funding for the work.

## Conflict of Interest Statement

The authors declare that the research was conducted in the absence of any commercial or financial relationships that could be construed as a potential conflict of interest.
