# Supplementary material for: Covert Infection of Insects by Baculoviruses
Source: Front Microbiol. 2017 Jul 17;8:1337. doi: 10.3389/fmicb.2017.01337 (PMC5511839; doi:10.3389/fmicb.2017.01337)
Supplement: Supplementary file 1 [file Table_1.pdf]

Supplemental Table 1. Summary of studies on baculovirus covert infection in natural and laboratory populations of insects over past 25 years.

Virus genus<br>Host/virus	Laboratory or natural population	Technique used for detection	Target gene(s)	Prevalence of infection	Reference
Alphabaculovirus					
Spodoptera exigua, SeMNPV	Laboratory	RT-PCR, qPCR	*DNA polymerase*	15-100% [(a)]<br>4% larvae [(b)]<br>15% adults [(b)]	Cabodevilla et al., 2011b
	Natural and laboratory	RT-PCR	*polyhedrin, DNA polymerase*	7-48% in wild adult males<br>1-38% in wild adult females [(c)]<br>16-100% in laboratory colony [(d)]	Cabodevilla et al., 2011a
	Natural	qPCR	*DNA polymerase*	51% in adult males<br>56% in adult females<br>22% in offspring [(e)]	Virto et al., 2014
	Laboratory	qPCR	*vp80*	5-56% in insects [(f)]	Murillo et al., 2011
	Natural	qPCR	*DNA polymerase*	50-90% in laboratory insects [(g)]<br>45-76% in adults from field [(g)]	Virto et al., 2017a
	Laboratory	qPCR	*DNA polymerase*	100% of adults in F_1 generation	Virto et al., 2017b
	Laboratory	qPCR	*DNA polymerase*	70-88% of adults	Carballo et al., 2017
Spodoptera exempta, SpexNPV	Laboratory	qPCR	*polyhedrin*	All stages positive [(h)]	Graham et al., 2015
	Natural and laboratory	nested PCR, nested RT-PCR	*polyhedrin, lef-8*	60-97% in wild adults [(i)]<br>50-90% in offspring F_1 [(i)]<br>39-93% in laboratory adults [(i)]	Vilaplana et al., 2010
	Natural	qPCR	*polyhedrin*	97% of larvae<br>100% of adult males	Graham et al., 2012
Spodoptera frugiperda SfMNPV	Laboratory	RT-PCR	*polyhedrin, ie-1*	5-19% in larvae [(j)]<br>0-26% in adults [(j)]	Martinez et al., 2005
	Laboratory	RT-PCR	*ie-0, sf29*	20 - 27% of larvae	Simón et al., 2010
Spodoptera spp. *SeMNPV, SfMNPV, SpliNPV*	Laboratory	RT-PCR	*ie-0, egt, DNA polymerase, chitinase, polyhedrin*	Positive results [(k)]	Simón et al., 2004
Mamestra brassicae MbMNPV	Laboratory	PCR, RT-PCR	*polyhedrin*	Positive result [(l)]	Hughes et al., 1993, 1997
	Natural and laboratory	RT-PCR	*polyhedrin*	50-100% in field collected insects<br>75-100% in laboratory reared offspring	Burden et al., 2003
	Laboratory	PCR - scintillation proximity assay	*polyhedrin*	Positive result [(m)]	Hughes et al., 1994

Supplemental Table 1, Williams et al. Frontiers in Microbiology 2017, doi: 10.3389/fmicb.2017.01337

Orgyia pseudotsugata OpMNPV	Laboratory	PCR	*polyhedrin* *op136*	6% in larvae [n]	Thorne et al., 2007
	Natural and laboratory	ELISA	n/a	0% in laboratory insects [o] 86-100% in field collected insects	Thorne et al., 2008
Operophtera brumata OpbuNPV	Natural	PCR	*polyhedrin*	0-40% in larvae [p] 10% in pupae	Burand et al., 2011
Spodoptera litura SpltNPV	Natural and laboratory	nested PCR	*lef-8*	1-47% in field collected insects 20% in laboratory colony	Kouassi et al., 2009
Choristoneura fumiferana CfMNPV, CfDEFNPV, GV	Natural and laboratory	multiplex PCR	*lef-8* *orf142* *granulin*	28% in laboratory insects [q] 71% in wild insects [q]	Kemp et al., 2011
Perina nuda PenuNPV	Laboratory	nested PCR	*polyhedrin*	92% of insects	Wang et al., 2000
Bombyx mori BmNPV	Laboratory	PCR	*polyhedrin*	Positive result	Ikuno et al., 2004
Lymantria dispar, LdMNPV	Laboratory	DNA hybridization	whole genome	Negative result (0%)	Murray et al., 1991
	Laboratory	PCR	*egt*	Positive result	Yang et al., 2015
	Natural	Dot-blot hybridization, PCR	*polyhedrin*	Positive result	Charpentier et al., 2003
	Natural and laboratory	PCR	*polyhedrin*	22% in pooled samples [r] 18 – 78% in offspring (F_1 and F_2)	Ilyinykh and Polenogova, 2013
Betabaculovirus					
Pieris brassicae PbGV	Laboratory	PCR	*polyhedrin*	Positive result	Sood et al., 2010
Cydia pomonella CpGV	Natural	PCR	not stated	0-15% of larvae [s]	Kundu et al., 2003
	Laboratory	PCR	*granulin*	40 – 94% of eggs and larvae [t]	Cossentine et al., 2005
	Natural	PCR	*granulin*	23-26% in larvae [u]	Eastwell et al., 1999
	Natural	PCR	*iap*	3 – 35% in larvae [v]	Arneodo et al., 2015
Tuta absoluta PhopGV	Natural	Dot-blot immunological test	n/a	0.7% of larvae [w]	Gómez-Valderrama et al., 2014
Plodia interpunctella PiGV	Laboratory	PCR, RT-PCR	*granulin*	30-100% (PCR) 40-100% (RT-PCR) 60-90% in offspring (RT-PCR) [x]	Burden et al., 2002

Deltabaculovirus					
Culex nigripalpus CuniNPV	Laboratory	Direct observation, bioassay	n/a	36% of adults	Becnel et al., 2003
Gammabaculovirus					
Neodiprion sertifer NeseNPV	Natural	qPCR	*vp39*	Positive result (y)	Krokene et al., 2013

(a) Prevalence of covert infection was dependent on concentration of inoculum consumed in larval stage.
(b) Average prevalence in third instar larvae and adults over five generations of laboratory rearing.
(c) Of the offspring produced by these insects, lethal virus disease was observed in 10 – 33% of offspring (PCR positive females), or 9-49% (PCR negative females).
(d) Prevalence varied with genotypic variant of SeMNPV.
(e) Two iflaviruses also present in infected insects.
(f) Six different insect lines tested, prevalence in pooled samples of various life stages. VP80 is a nucleocapsid protein. MbMNPV was also detected in some insect lines.
(g) Prevalence of covert infection varied between genotypic variants; 20-31% of control insects also infected.
(h) Evidence of persistent covert infection detected in all life stages; virus load varies according to stage and tissue.
(i) RT-PCR consistently provided a lower estimate of the prevalence of covert infection than conventional PCR.
(j) Larvae were analyzed in the fifth instar. Prevalence varied according to PCR amplified gene. The *ie-1* transcripts were not amplified from adults.
(k) Results varied according to host - virus combination and temporal expression of genes in hemocoel, midgut and whole insect.
(l) The *ie-1* and *p6.9* genes also shown to be transcriptionally active by transient assay.
(m) Scintillation proximity assay used to estimate number of viral genome copies in fat body of covertly infected insects.
(n) Sensitivity of PCR compared to PCR + Southern hybridization and ELISA; PCR results had higher virus specificity with *op136* compared to *polyhedrin.*
(o) No covert infections were detected following an initial peak of mortality in laboratory-inoculated insects.
(p) Prevalence varied between collection sites.
(q) Three baculoviruses present in natural and laboratory insects. CfMNPV was the most prevalent virus but mixed infections were also detected.
(r) Embryos dissected from field-collected eggs were pooled in groups of 20 embryos for PCR analysis.
(s) Only 1 out of 17 collections over four years were positive for covertly infected insects
(t) Prevalence of covert infection increased during immature stages.
(u) Prevalence given as mean values at each location (prevalence ranged between 0 and 50% within sites).
(v) Mean prevalence at two locations where virus had been applied in the past. Prevalence at sites that had not been treated with virus was zero.
(w) The identity of the granulovirus in insects was not identified but it showed immunological reactivity to PhopGV antisera
(x) Both sexes participated in vertical transmission to offspring; eggs and larvae were tested.
(y) Prevalence of covert infection of larvae not stated; study aimed to determine limits for detection of viral DNA in insects and environmental samples. The *vp39* gene encodes a viral capsid protein.

REFERENCES

Arneodo, J. D., De Anna, J., Salvador, R., Farinon, M., Quintana, G. and Sciocco-Cap, A. (2015). Prospection and molecular analysis of CpGV isolates infecting *Cydia pomonella* at different geographical locations in Argentina. *Ann. Appl. Biol.* 166, 67-74.

Becnel, J. J., White, S. E., and Shapiro, A. M. (2003). *Culex nigripalpus* nucleopolyhedrovirus (CuniNPV) infections in adult mosquitoes and possible mechanisms for dispersal. *J. Invertebr. Pathol.* 83, 181-183.

Burand, J., Kim, W., Welch, A., and Elkinton, J. S. (2011). Identification of a nucleopolyhedrovirus in winter moth populations from Massachusetts. *J. Invertebr. Pathol.* 108, 217-219.

Burden, J. P., Griffiths, C. M., Cory, J. S., Smith, P., and Sait, S. M. (2002). Vertical transmission of sublethal granulovirus infection in the Indian meal moth, *Plodia interpunctella*. *Mol. Ecol.* 11, 547-555.

Burden, J. P., Nixon, C. P., Hodgkinson, A. E., Possee, R. D., Sait, S. M., King, L. A. et al. (2003). Covert infections as a mechanism for long-term persistence of baculoviruses. *Ecol. Lett.* 6, 524-531.

Cabodevilla, O., Ibañez, I., Simón, O., Murillo, R., Caballero, P., and Williams, T. (2011a). Occlusion body pathogenicity, virulence and productivity traits vary with transmission strategy in a nucleopolyhedrovirus. *Biol. Control* 56, 184-192.

Cabodevilla, O., Villar, E., Virto, C., Murillo, R., Williams, T., and Caballero, P. (2011b) Intra- and intergenerational persistence of an insect nucleopolyhedrovirus: adverse effects of sublethal disease on host development, reproduction, and susceptibility to superinfection. *Appl. Environ. Microbiol.* 77, 2954-2960.

Carballo, A., Murillo, R., Jakubowska, A., Herrero, S., Williams, T., and Caballero, P. (2017). Co-infection with iflaviruses influences the insecticidal properties of Spodoptera exigua multiple nucleopolyhedrovirus occlusion bodies: implications for the production and biosecurity of baculovirus insecticides. *PLoS ONE* 12, e0177301.

Charpentier, G., Desmarteaux, D., Bourassa, J. P., Belloncik, S., and Arella, M. (2003). Utilization of the polymerase chain reaction in the diagnosis of nuclear polyhedrosis virus infections of gypsy moth (*Lymantria dispar*, Lep., Lymantriidae) populations. *J. Appl. Entomol.* 127, 405-412.

Cossentine, J. E., Jensen, L. B. M., and Eastwell, K. C. (2005). Incidence and transmission of a granulovirus in a large codling moth [*Cydia pomonella* L.(Lepidoptera: Tortricidae)] rearing facility. *J. Invertebr. Pathol.* 90, 187-192.

Eastwell, K. C., Cossentine, J. E., and Bernardy, M. G. (1999). Characterisation of *Cydia pomonella* granulovirus from codling moths in a laboratory colony and in orchards of British Columbia. *Ann. Appl. Biol.* 134, 285-291.

Gómez-Valderrama, J., Herrera, L., Uribe-Vélez, D., López-Ferber, M., and Villamizar, L. (2014). An immunological method for granulovirus detection in larvae of *Tuta absoluta*: searching for isolates with prospects for biological control of this pest in Colombia. *Int. J. Pest Manag.* 60, 136-143.

Graham, R. I., Grzywacz, D., Mushobozi, W. L., and Wilson, K. (2012). *Wolbachia* in a major African crop pest increases susceptibility to viral disease rather than protects. *Ecol. Lett*. 15, 993-1000.

Graham, R. I., Tummala, Y., Rhodes, G., Cory, J. S., Shirras, A., Grzywacz, D., et al. (2015). Development of a real-time qPCR assay for quantification of covert baculovirus infections in a major African crop pest. *Insects* 6, 746-759.

Hughes, D. S., Possee, R. D., and King, L. A. (1993). Activation and detection of a latent baculovirus resembling *Mamestra brassicae* nuclear polyhedrosis virus in *M. brassicae* insects. *Virology* 194, 608-615.

Hughes, D. S., Possee, R. D., and King, L. A. (1994). Quantification of latent *Mamestra brassicae* nuclear polyhedrosis virus in *M. brassicae* insects using a PCR-scintillation proximity assay. *J. Virol. Meth.* 50, 21-27.

Hughes, D. S., Possee, R. D., and King, L. A. (1997). Evidence for the presence of a low-level, persistent baculovirus infection of *Mamestra brassicae* insects. *J. Gen. Virol.* 78, 1801-1805.

Ikuno, A. A., Margatho, L. F. F., Harakava, R., Akamatsu, M. A., Martins, E. M. F., Porto, A. J. et al. (2004). Direct application of the new PCR protocol for evaluation and monitoring of *Bombyx mori* infection by nucleopolyhedrovirus. *Arq. Inst. Biol.* 71, 309-315.

Ilyinykh, A. V., and Polenogova, O. V. (2013). Demonstration of remote effect for vertical transmission of baculovirus based on example of gypsy moth, *Lymantria dispar* L. (Lepidoptera, Lymantriidae). *Biol. Bull. Rev.* 3, 214-218.

Kemp, E. M., Woodward, D. T., and Cory, J. S. (2011). Detection of single and mixed covert baculovirus infections in eastern spruce budworm, *Choristoneura fumiferana* populations. *J. Invertebr. Pathol.* 107, 202-205.

Kouassi, L. N. G., Tsuda, K., Goto, C., Mukawa, S., Sakamaki, Y., Kusigemati, K. et al. (2009). Prevalence of latent virus in *Spodoptera litura* (Fabricius) (Lepidoptera: Noctuidae) and its activation by a heterologous virus. *Appl. Entomol. Zool.* 44, 95-102.

Krokene, P., Heldal, I., and Fossdal, C. G. (2013). Quantifying *Neodiprion sertifer* nucleopolyhedrovirus DNA from insects, foliage and forest litter using the quantitative real-time polymerase chain reaction. *Agric. Forest Entomol.* 15, 120-125.

Kundu, J. K., Stará, J., Kocourek, F., and Pultar, O. (2003). Polymerase chain reaction assay for *Cydia pomonella* granulovirus detection in *Cydia pomonella* population. *Acta Virol.* 47, 153-158.

Martínez, A. M., Williams, T., López-Ferber, M., and Caballero, P. (2005). Optical brighteners do not influence covert baculovirus infection of *Spodoptera frugiperda*. *Appl. Environ. Microbiol.* 71, 1668-1670.

Murillo, R., Hussey, M. S., and Possee, R. D. (2011). Evidence for covert baculovirus infections in a *Spodoptera exigua* laboratory culture. *J. Gen. Virol.* 92, 1061-1070.

Murray, K. D., Shields, K. S., Burand, J. P., and Elkinton, J. S., 1991. The effect of gypsy moth metamorphosis on the development of nuclear polyhedrosis virus infection. *J. Invertebr. Pathol.* 57, 352-361.

Simón, O., Williams, T., López-Ferber, M., and Caballero, P. (2004). Genetic structure of a *Spodoptera frugiperda* nucleopolyhedrovirus population: high prevalence of deletion genotypes. *Appl. Env. Microbiol.* 70, 5579-5588.

Simón, O., Williams, T., Possee, R. D., López-Ferber, M., and Caballero, P. (2010). Stability of a *Spodoptera frugiperda* nucleopolyhedrovirus deletion recombinant during serial passage in insects. *Appl. Env. Microbiol.* 76, 803-809

Sood, P., Mehta, P. K., Bhandari, K., and Prabhakar, C. S. (2010). Transmission and effect of sublethal infection of granulosis virus (PbGV) on *Pieris brassicae* Linn. (Pieridae: Lepidoptera). *J. Appl. Entomol.* 134, 774-780.

Thorne, C. M., Otvos, I. S., Conder, N. and Levin, D. B. (2007). Development and evaluation of methods to detect nucleopolyhedroviruses in larvae of the Douglas-fir tussock moth, *Orgyia pseudotsugata* (McDunnough). *Appl. Env. Microbiol.* 73, 1101-1106.

Thorne, C. M., Levin, D. B., Otvos, I. S., and Conder, N. (2008). Virus loads in Douglas-fir tussock moth larvae infected with the *Orgyia pseudotsugata* nucleopolyhedrovirus. *Can. Entomol.* 140, 158-167.

Vilaplana, L., Wilson, K., Redman, E., and Cory, J. (2010). Pathogen persistence in migratory insects: high levels of vertically-transmitted virus infection in field populations of the African armyworm. *Evol. Ecol.* 24, 147-160.

Virto, C., Navarro, D., Tellez, M. M., Murillo, R., Williams, T., and Caballero, P. (2017b). The role of chemical and biological stress factors on the activation of nucleopolyhedrovirus infections in covertly infected *Spodoptera exigua* larvae. *J. Appl. Entomol.* 141, 384–392.

Virto, C., Navarro, D., Tellez, M.M., Herrero, S., Williams, T., Murillo R. et al. (2014). Natural populations of *Spodoptera exigua* are infected by multiple viruses that are transmitted to their offspring. *J. Invertebr. Pathol.* 122, 22-27.

Virto, C., Williams, T., Navarro, D., Tellez, M.M., Murillo, R., and Caballero, P. (2017a). Can mixtures of horizontally and vertically transmitted nucleopolyhedrovirus genotypes be effective for biological control of *Spodoptera exigua*? *J. Pest Sci.* 90, 331-343.

Wang, C. H., Yang, H. N., Liu, H. C., Kou, G. H. and Lo, C. F. (2000). Nested polymerase chain reaction and in situ hybridization for detection of nucleopolyhedrosis. *J. Virol. Meth.* 84, 65-75.

Yang, M. M., Zhai, W. J., Li, B. C., Zhang, W., Zhang, Z. Q., Li, M. L. et al. (2015). Covert LdMNPV detected in *Lymantria dispar* larvae during a survey of potential hosts for DekiNPV production. *Int. J. Pest Manag.* 61, 26-29.
